# Prior physical exertion modulates allocentric distance perception: a demonstration of task-irrelevant cross-modal transfer

**DOI:** 10.1007/s00221-016-4641-5

**Published:** 2016-04-06

**Authors:** Ella V. Clark, Nick S. Ward, Annapoorna Kuppuswamy

**Affiliations:** Institute of Neurology, University College London, 33, Queen Square, London, WC1N 3BG UK

**Keywords:** Perception, Distance estimation, Physical exertion, Allocentric

## Abstract

Physical exertion has been previously shown to influence distance perception in the egocentric framework. In this study, we show that physical exertion influences allocentric distance perception. Twenty healthy volunteers made allocentric line length estimates following varying levels of physical exertion. Each participant was presented with 30 different line lengths ranging from 1 to 12 cm, and each length was presented three times. Each line presentation was preceded by the participant exerting one of the following three levels of their maximal voluntary force (MVF): 20, 50, or 80 % MVF using their hand in the pinch force task. Psychometric curves were obtained for the lines perceived as ‘long’ following each of the three force levels. Lines that were perceived as ‘short’ following 20 and 50 % MVF were perceived as ‘long’ following 80 % MVF; that is, there was a significant leftward shift in the psychometric curve following 80 % MVF when compared to 20 and 50 % MVF. Here, we demonstrate that physical exertion influences perception of distances in the allocentric framework. We discuss our findings with respect to cross-modal interactions, fatigue physiology, peri- and extra-personal space interactions.

## Introduction

Our ability to judge distances or spatial magnitude estimation is influenced by several factors, one of which is the possibility of movement in the given space. For example, our ability to judge the distance between ourselves and a piece of cake is influenced by whether it is within our reach or if there are barriers between ourselves and the goal. The possibility of movement is determined by the functional capability and physiological status of the individual.

In experimental paradigms, the physiological status of the individual is altered by repetition of an action. Sustained repetition of a physical task induces fatigue in both the peripheral musculature (Barry and Enoka 2007; Enoka and Duchateau 2008; Enoka and Stuart 1992) and central neural components (Taylor et al. 2006; Todd et al. 2003) that drive action, leading to a drop in performance. Not only is the performance affected, but there are also perceptual consequences to sustained repetition which manifests as an increase in performance-related effort (Lampropoulou and Nowicky 2014; Sacco et al. 1999; Scotland et al. 2014) eventually leading to the feeling of fatigue. Studies also show that the increase in perceived effort systematically covaries with movement-related cortical potentials and motor excitability (Slobounov et al. 2004; Takarada et al. 2014), suggesting that alterations in effort perception is directly linked to movement.

Cross-modal interactions in perception are very common, and it is important for adaptive behaviour. The interaction between movement-related perceived effort and spatial magnitude estimation has been studied extensively; for example, a distance is estimated to be longer if one is asked to carry a heavy weight while scaling the distance as opposed to when walking without a weight (Bloesch et al. 2012; Cardellicchio et al. 2013; Costantini et al. 2011; Sugovic and Witt 2013; Witt and Proffitt 2008; Witt et al. 2005). Most paradigms require egocentric (with respect to self) estimation of distances. In such egocentric paradigms, perception of effort is directly relevant to the task (such as to scale the distance). Recently, it was proposed that task-irrelevant perceptual value in one sensory modality influenced task performance involving another sensory modality (Pooresmaeili et al. 2014). For example, reward association in an auditory task increased visual acuity if the reward-related tone was presented during performance of a visual perception task even though the reward value associated with the auditory tone was irrelevant to the visual perception task.

We set out to study if such cross-modal transfer can be observed in perceptual changes generated by internal stimuli relating to the physiological status of the individual (such as sense of effort), when interacting with an external stimuli, such as vision. We investigated if performing a high-effort task prior to the estimation of a line length (allocentric spatial magnitude estimation task) leads to differences in line length estimation following a low-effort task.

## Methods

Twenty healthy young adults (age range 22–40) participated in the study following informed consent. The study was approved by the Riverside Research Ethics Committee (12/LO/1474). All participants attended a single laboratory session and were in good health and alert while participating in the experiment.

Force calibration and force task: Participants were asked to grip a pinch force meter (Biometrics F100) with their right index and thumb and apply their maximum effort. This procedure was repeated three times. The output from the pinch force meter was visualised using Spike software. The average of the three attempts was taken as that individual’s maximum force level. In the force task, participants gripped the pinch force meter for 5 s. The participants were given visual feedback of the force levels produced during grip. The participants had to produce one of the following three force levels: 20, 50, or 80 % of their maximum force level.

### Line length familiarisation

Participants were shown six lines of lengths: three that belonged to the ‘short’ category—1, 2, and 3 cm—and three to the ‘long’ category—10, 11, and 12 cm. When each line was presented, the participants were told if the presented line was considered ‘short’ or ‘long’. This was repeated several times until the participants were able to distinguish between the ‘shorts’ and the ‘longs’.

### Experimental set-up

Participants were seated comfortably with the pinch force meter held in their right hand. Two monitors were placed in front of the participant (approximately 25 in. in front of the participant): one provided visual feedback of the applied force level and the other presented the participant with lines of different lengths.

### Experimental protocol

The experiment consisted of three blocks of 30 trials each. Each trial consisted of a 5-s pinch grip task followed by estimation of line length. Participants applied one of the following three forces: 20, 50, or 80 % MVF, and 30 different lines were presented with lengths ranging from 1 to 12 cm. Of the 30 lines, six had already been presented in the familiarisation block. A line of a given length was presented three times, each preceded by a different force level. A given force and line length combination was presented only once making a total of 90 trials. The order of forces and line lengths was randomised with equal numbers of the three different force levels in each block. Participants were asked to report if the presented line was ‘short’ or ‘long’. They were instructed to base their estimation on the length of lines presented during the familiarisation phase. If they determined the presented line to be shorter than half the longest line presented during familiarisation, they reported ‘short’, and if the line was estimated to be longer than half the longest line presented, they reported ‘long’.

### Analysis

To identify if prior physical exertion had any influence on line length estimation, the data for each individual were divided based on force level. For every line length, the total number of ‘long’ responses across the 20 individuals was counted. As the expected distribution of the total ‘long’ responses across the line lengths starting from the shortest to the longest was sigmoidal, a sigmoidal curve fitting procedure was performed for each of the three force levels. The equation used was *f* = *a*/(1 + exp(−(*x*−*x*0)/*b*)), *x*0 = *x*50(*x*,*y*,.5); *x*0 here represents the group estimate of the mid-way point of the longest line presented (12 cm) at each of the three force levels.

## Results

All participants were able to distinguish between short and long lines at the end of familiarisation phase.

**Fig. 1:**
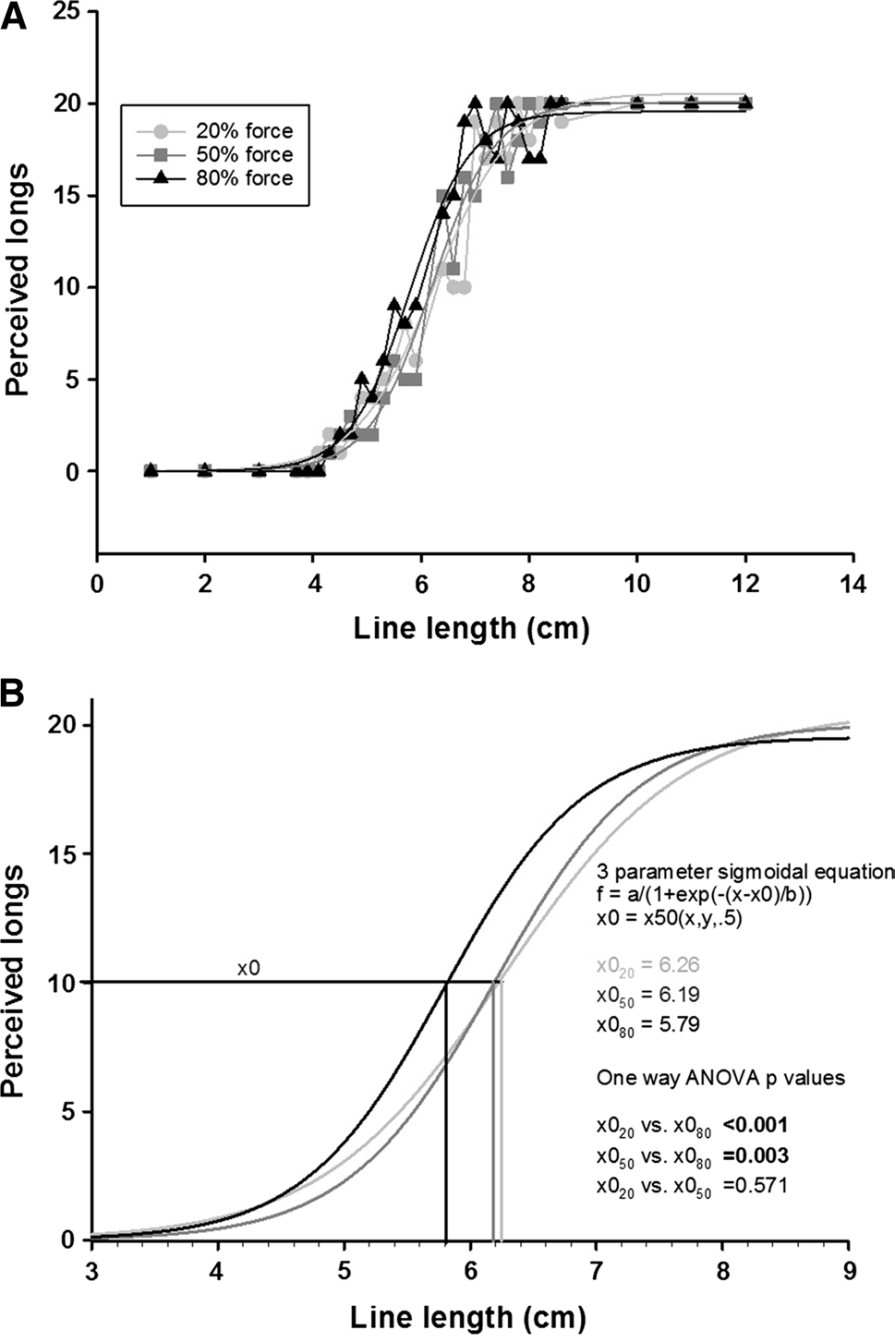
In **a**, the total number of ‘long’ responses is shown on the *y-*axis while the length of the presented line is shown on the *x-*axis. The *light grey circular symbols* represent the responses that were given following 20 % force production, the *dark grey square symbols* represent responses following 50 % force production, and *black triangular symbols* represent those following 80 % force production. In **b**, the fitted psychometric curves of the three force levels are shown. A significant difference is seen between the 80 and 20 % curves and likewise with 50 % curves

Figure 1a shows the distribution of ‘long’ responses for every length presented. This figure illustrates that all participants correctly categorised the six line lengths presented during familiarisation phase irrespective of the preceding force applied. However, the lengths in between were variously reported as being ‘long’ or ‘short’ and were influenced by the preceding force levels.

The estimated mid-point of the 12-cm line following 20 % force exertion was 6.26 cm, 50 % force exertion was 6.19 cm, and 80 % force exertion was 5.79 cm. A one-way ANOVA between the numbers of ‘long’ estimates at each force level revealed a significant difference between the 20 and 80 % conditions (*p* < 0.001) and 50 and 80 % conditions (*p* = 0.003) (Fig. 1b).

## Discussion

The main finding of this study is a significant increase in the perceived length of a line when the estimation is preceded by high-level physical exertion compared to the estimation following low-level physical exertion. To our knowledge, this is the first demonstration of task-irrelevant cross-modal interaction between internally generated perception (effort) and externally driven perceptual process (visual perception), and here we discuss the implications of this finding.

There is increasing interest in understanding cross-modal interactions, and the vast majority of studies address the interaction between perceptual processes that are driven by external stimuli, i.e. auditory and visual sensory modalities (Bruns et al. 2014; Eimer et al. 2004; Komura et al. 2005; Pooresmaeili et al. 2014). An emerging mechanistic principle of perceptual cross-modal transfer is that reward association in a task within a modality influences task performance in another modality even if the reward association did not apply to the other modality (Bruns et al. 2014; Pooresmaeili et al. 2014). The current study suggests that perception driven by internal stimuli (physiological state of the individual) may interact similarly with externally driven perception (visual perception). The systematic influence of perceived effort on line length estimation, a visual perception task for which effort is irrelevant, supports the proposed above principle of cross-modal transfer. There is extensive evidence for the physiological state of the individual influencing task performance. In some studies, the physiological state is directly relevant to the task performed (Feeney et al. 2015), while in others, such as in dual-task paradigms (Watanabe and Funahashi 2015; Wollesen et al. 2016), despite tasks being unrelated, performance in both tasks deteriorates, normally attributed to increased cognitive load or shared neural processing. The current result suggests that perceptual distortion may be a possible mechanism by which alteration of effort influences dual-task paradigms.

In this study, the visual perception task was presented within the participant’s peri-personal space. Although the boundaries of peri-personal space differ based on context, experience, and individual differences (Serino 2016), here we define it as space within the arm’s reach. Cross-modal transfer is thought to be stronger within the peri-personal space (Van der Biest et al. 2016). Interaction between physiological state and distance perception in the peri-personal space is greatly influenced by whether or not one is able to reach the object whose distance of separation one is estimating (Witt et al. 2004). The same applies to extra-personal space distance estimation: if one is able to easily get to the object from which they are separated or physiological state allows easy scaling of a required distance, it appears closer/shorter. Most studies use egocentric paradigms, i.e. distance estimation with respect to self. However, true cross-modal transfer can only be inferred when there is task-irrelevant transfer. Allocentric distance estimation paradigm is one such where physiological state of the observer is irrelevant to distance estimation. A recent study observed that even in allocentric distance judgements, the potential for movement influenced distance estimation (Fini et al. 2015); for example, distance between two apples is estimated to be longer in comparison with distance between a man and an apple despite the actual distance between the objects being the same. In our study we show that, allocentric spatial judgements when in the peri-personal space, is influenced by the internal state of the perceiver. It is yet to be seen if this task-irrelevant transfer holds true in the extra-personal space.

Fatigue is extremely common in both health and disease (Chaudhuri and Behan 2004). The definition of fatigue is context specific and can refer to both decrease in performance and the perception of fatigue. While performance decrease is easily quantifiable, perceptual fatigue is harder to define and quantify, and does not linearly map on to performance. However, perceptual fatigue and effort perception are more directly correlated than with task performance (Lampropoulou and Nowicky 2014; Sacco et al. 1999; Thickbroom et al. 2006; Wallman and Sacco 2007). Emerging evidence suggests that chronic neurological fatigue may be a disorder of effort perception (Kuppuswamy et al. 2015) although it is unclear what triggers alterations in effort perception. If altered effort perception influences visual perception, can the reverse be true? It may be that alteration in effort perception may be triggered by alteration in another perceptual domain such as vision. Quantifying perceptual fatigue (Johansson et al. 2014; Lerdal et al. 2011) is fraught with problems, partly a problem of reporting bias. Cross-modal transfer of fatigue/effort provides an opportunity to develop an irrelevant task which is influenced by fatigue/effort and mitigate the problem of reporting bias.

In summary, we have demonstrated that cross-modal transfer can be observed between internally generated perceptual state and externally driven perceptual process. This result has implications for our understanding of perceptual cross-modal transfer, fatigue physiology, and peri- and extra-personal space interaction.
